# Sp1 and c-Myc modulate drug resistance of leukemia stem cells by regulating survivin expression through the ERK-MSK MAPK signaling pathway

**DOI:** 10.1186/s12943-015-0326-0

**Published:** 2015-03-07

**Authors:** Yi Zhang, Hai-xuan Chen, Shu-yan Zhou, Shao-xiang Wang, Kai Zheng, Dan-dan Xu, Yu-ting Liu, Xiao-yan Wang, Xiao Wang, Hai-zhao Yan, Li Zhang, Qiu-ying Liu, Wan-qun Chen, Yi-fei Wang

**Affiliations:** College of Life Science and Technology, Jinan University, 510632 Guangzhou, P.R China; Institute of Biomedicine, Jinan University, 510632 Guangzhou, P.R China; Department of Pathological Physiology, Wan-nan Medical College, 241000 Wuhu, P.R China; College of Medicine, Jinan University, 510632 Guangzhou, P.R China; College of Medicine, Shenzhen University, 518020 Shenzhen, P.R China

**Keywords:** Survivin, Leukemia stem cell, Sp1, c-Myc, ERK, MSK pathway

## Abstract

**Background:**

Acute myeloid leukemia (AML) is initiated and maintained by a subset of self-renewing leukemia stem cells (LSCs), which contribute to the progression, recurrence and therapeutic resistance of leukemia. However, the mechanisms underlying the maintenance of LSCs drug resistance have not been fully defined. In this study, we attempted to elucidate the mechanisms of LSCs drug resistance.

**Methods:**

We performed reverse phase protein arrays to analyze the expression of anti-apoptotic proteins in the LSC-enriched leukemia cell line KG-1a. Immuno-blotting, cell viability and clinical AML samples were evaluated to verify the micro-assay results. The characteristics and transcriptional regulation of survivin were analyzed with the relative luciferase reporter assay, mutant constructs, chromatin immuno-precipitation (ChIP), quantitative real-time reverse transcription polymerase chain reaction (RT-qPCR), and western blotting. The levels of Sp1, c-Myc, phospho-extracellular signal-regulated kinase (p-ERK), phospho-mitogen and stress-activated protein kinase (p-MSK) were investigated in paired CD34+ and CD34- AML patient samples.

**Results:**

Survivin was highly over-expressed in CD34 + CD38- KG-1a cells and paired CD34+ AML patients compared with their differentiated counterparts. Functionally, survivin contributes to the drug resistance of LSCs, and Sp1 and c-Myc concurrently regulate levels of survivin transcription. Clinically, Sp1 and c-Myc were significantly up-regulated and positively correlated with survivin in CD34+ AML patients. Moreover, Sp1 and c-Myc were further activated by the ERK/MSK mitogen-activated protein kinase (MAPK) signaling pathway, modulating survivin levels.

**Conclusion:**

Our findings demonstrated that ERK/MSK/Sp1/c-Myc axis functioned as a critical regulator of survivin expression in LSCs, offering a potential new therapeutic strategy for LSCs therapy.

**Electronic supplementary material:**

The online version of this article (doi:10.1186/s12943-015-0326-0) contains supplementary material, which is available to authorized users.

## Background

Acute myeloid leukemia (AML) is an aggressive malignancy with a-5 year overall survival rate of approximately 30%-45% in adults and nearly 70% in children [1]. Although the survival rate in children is quite high, recurrence and acquired drug-resistance remain the leading causes of death, occurring in over 60% of cases by 1 year after complete remission [2]. AML is typically a stem cell-driven disease, and the existence of leukemia stem cells (LSCs) was first identified (CD34 + CD38-) by JE Dick in 1994; these cells extremely promote AML progression, chemo-resistance and recurrence [3,4]. Therefore, identifying molecules or pathways that are preferentially expressed in LSCs and determining the principles controlling AML pathogenesis are critical and may facilitate the optimization of clinical treatments for AML patients.

In recent years, many researchers have focused on the development of novel therapies targeting cancer stem cells (CSCs), including specific signaling pathways, the tumor microenvironment, and induction of differentiation and apoptosis [5]. From these studies, survivin has emerged as an essential factor for tumor progression and development [6,7]. Survivin, a member of the inhibitors of apoptosis (IAP) protein family, is one of the most frequently elevated targets in numerous malignancies and is largely undetectable or expressed at very low levels in normal and adult tissues. Moreover, survivin has been linked with poor prognosis, resistance to therapy, and low overall survival [8]. Additionally, survivin is localized in both the cytoplasm and nucleus and has an active nuclear transporter in its linker region [9]. Cytoplasmic survivin co-operatively interacts with XIAP/Smac/DIABLO to regulate the caspase cascade function by directly preventing its release and activation from mitochondria for the induction of apoptosis [10]. Nuclear survivin predominantly associates with the Aurora B kinase/INCENP/Borealin/Dasra B complex to form a chromosomal passenger, which regulates the progression of the cell-cycle from the centromere stage to the mitotic spindle stage during cell division [11,12]. Moreover, survivin has been confirmed to function as an essential factor in the tumor microenvironment and in multiple signaling pathways, including PI3K, Akt, p53, NF-kB, STAT3, Wnt/β-catenin and MAPK, which are highly involved in controlling tumor maintenance and growth promotion [13-15].

Survivin has also been shown to be associated with drug resistance in various cancers or tissues. Park et al showed that survivin is up-regulated in primary acute lymphoblastic leukemia (ALL) and plays a critical role in drug resistance, and inhibition of survivin expression with chemotherapy leads to the eradication of ALL [16]. Fukuda reported that silencing survivin could significantly reduce the proliferation of leukemia cells and induce apoptosis in FLT3 mutant mice [17]. Intriguingly, survivin expression is correlated with Oct-4 expression, allowing concurrently regulation of murine embryonic stem cell survival under stress [18], demonstrating a novel function for controlling the stem cell state. These results suggest that survivin is a potentially worthy target for personalized molecular therapy. However, the effects of survivin on the regulating of CSC biological properties remains to be defined.

In the current study, we aimed to investigate the role of survivin in LSCs drug resistance and to elucidate the mechanisms regulating survivin expression in LSCs. Our results demonstrated that the ERK-MSK-specificity protein (Sp) 1/c-Myc axis was responsible for survivin expression, providing valuable targets for further development of molecular therapies to treat leukemia.

## Results

### Survivin is highly expressed in LSCs compared with their differentiated counterparts

Several recent studies have suggested that as much as 25% of cancer cell lines and certain tumor samples have the properties of CSCs [19]. Therefore, we first isolated LSCs using magnetic sorting with the gold standard surface markers CD34 + CD38- from six leukemia cell lines (Additional file 1: Figure S1A). KG-1a and MOLM13 cells were enriched in LSCs (9.6% and 4.3%, respectively, Additional file 1: Figure S1B). Subsequently, we identified the isolated LSCs by examination of typical stem cell gene expression, cell surface marker ABCB1 (P-gp), which was preferentially expressed in LSCs [20,21], self-renewal ability, and drug resistance (using three first-line AML drugs, Ara-C, Dexamethasone and L-aspartic acid). This analysis suggested that these cells were indeed LSCs and could be used for further studies (Additional file 1: Figure S1 and Additional file 2: Figure S2A).

Next, to demonstrate the specific molecular mechanisms underlying the chemo-resistance of LSCs, we used the ABC transporter inhibitor verapamil [22] to investigate the dependence of LSCs drug resistance on the expression of the “drug pump”. As expected, the addition of verapamil increased Ara-C and dexamethasone-induced apoptosis (Additional file 2: Figure S2B). However, the LSCs fractions still showed reduced apoptosis and higher resistance to chemotherapeutic agents than non-LSCs subpopulations in KG-1a cells (Additional file 2: Figure S2C). Therefore, we speculated that there might be other mechanisms mediating drug-resistance in LSCs.

**Figure 1:**
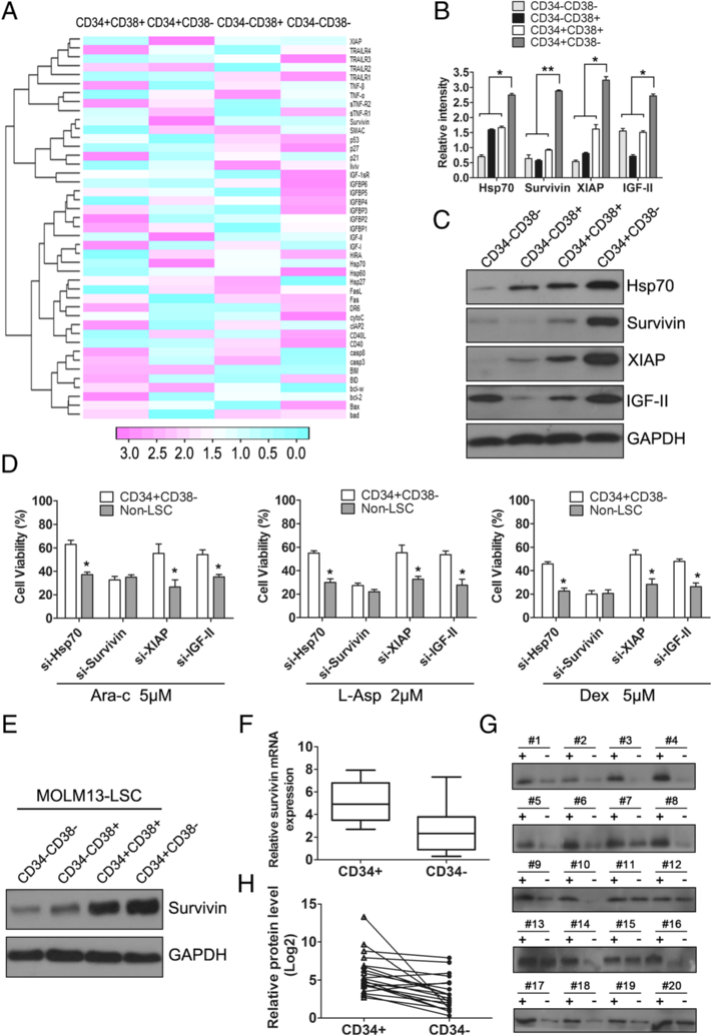
**Survivin was highly expressed in leukemia stem cells compared to their differentiated counterparts. (A)** KG-1a cells were separated into four subpopulations, and apoptosis-related protein micro-assays were performed using R statistical programming. **(B)** The relative expression level of four apoptosis-related proteins (Hsp70, survivin, XIAP, and IGF-II) were determined (^*^ *P* < 0.05, ^**^ *P* < 0.01). **(C)** Protein assay results were confirmed by western blotting. **(D)** LSCs and non-LSCs viability was analyzed following inhibition of survivin, Hsp70, XIAP, or IGF-II expression with siRNA in the presence of chemical agents (Ara-C, L-Asp, and Dexamethasone [Dex]). **(E)** Analysis of survivin expression in MOLM13 cells following fractionation for the CD34 + CD38- population. **(F)** Survivin mRNA expression was analyzed in CD34+/CD34- cells from AML patients (n = 56, *P < 0.05) by real-time qPCR. **(G)** Survivin protein expression was analyzed in paired CD34+/CD34- samples from 20 AML patients by western-blot. (H) Line graph showing a direct comparison of survivin protein levels in CD34+/CD34- cells from paired AML samples (n = 20, ^*^ *P* < 0.05).

To investigate whether anti-apoptosis-related proteins were involved in LSCs drug resistance, we performed reverse-phase protein microarray (RPPA) to analyze the expression of apoptosis-related proteins in the four populations (CD34 + CD38+, CD34 + CD38-, CD34-CD38+, CD34-CD38-) isolated from KG-1a cells (Figure 1A and Additional file 3: Figure S3A). Intriguingly, we found that four apoptosis-related proteins, i.e., Hsp70, survivin, XIAP, and insulin-like growth factor (IGF)-II, were highly expressed in CD34 + CD38- cells compared with other subsets (Figure 1B). Immuno-blotting analysis yielded similar results as the protein microarray data in KG-1a cells (Figure 1C). To further investigate which specific proteins mediated the drug resistance of LSCs, we used specific short-interfering RNAs (siRNAs). As shown in Figure 1D, only inhibition of survivin expression decreased the viability of LSCs consistent with the observed effects in non-LSCs following addition of Ara-C, L-Asp, and Dexamethasone, indicating that survivin could block chemotherapy-induced apoptosis. Additionally, survivin was also found to be highly expressed in the LSC-enriched fraction of MOLM13 cells (Figure 1E).

To determine whether up-regulation of survivin expression in CD34 + CD38- cell fractions was specific for LSCs, we collected blood samples from 56 patients with AML to directly compare survivin levels in paired CD34+ and CD34- cells. Using RT-qPCR, survivin was found to be significantly up-regulated in primary CD34+ cells from AML patients (89.3%, 50/56) when compared with their corresponding CD34- counterparts (*P* < 0.05, Figure 1F). Accordingly, direct comparison of survivin protein levels in 20 paired CD34+ and CD34- samples showed similar trends for survivin mRNA expression (Figure 1G-1H). In addition, we found no obvious relationship between survivin levels and any of the following clinical characteristics: age, sex, French-American-British (FAB) subtype, karyotype, white blood cells, platelets, bone marrow (BM) or peripheral blood (PB) blast counts, and hemoglobin levels (Table 1).

**Table 1:** **Demographics and clinical characteristics of 56 newly diagnosed AML patients in this study**

**Characteristics (n)**	**Survivin CD34+ expression, n (%)**	**CD34- expression, n (%)**
Sex		
Female (17)	8 (47%)	9 (53%)
Male (39)	23 (59%)	16 (41%)
*P* value	0.342	
Age		
≤40 years old (30)	19 (63%)	11 (37%)
>40 years old (24)	10 (42%)	14 (58%)
*P* value	0.226	
FAB		
M0 (5)	3 (60%)	2 (40%)
M1 (12)	10 (83%)	2 (17%)
M2 (8)	2 (25%)	6 (75%)
M5 (23)	17 (74%)	6 (26%)
M7 (8)	1 (13%)	7 (87%)
*P* value	0.148	
Laboratory analysis (median)		
WBC (10^9^/L)	16.4	15.8
Platelet (10^9^/L)	87	106
Hemoglobin (g/dL)	9.6	4.3
BM blast, %	36	21
PB blast, %	17	10
*P* value	0.174	

### Survivin contributed to the anti-apoptotic and chemoresistant characteristics of LSCs

**Figure 2:**
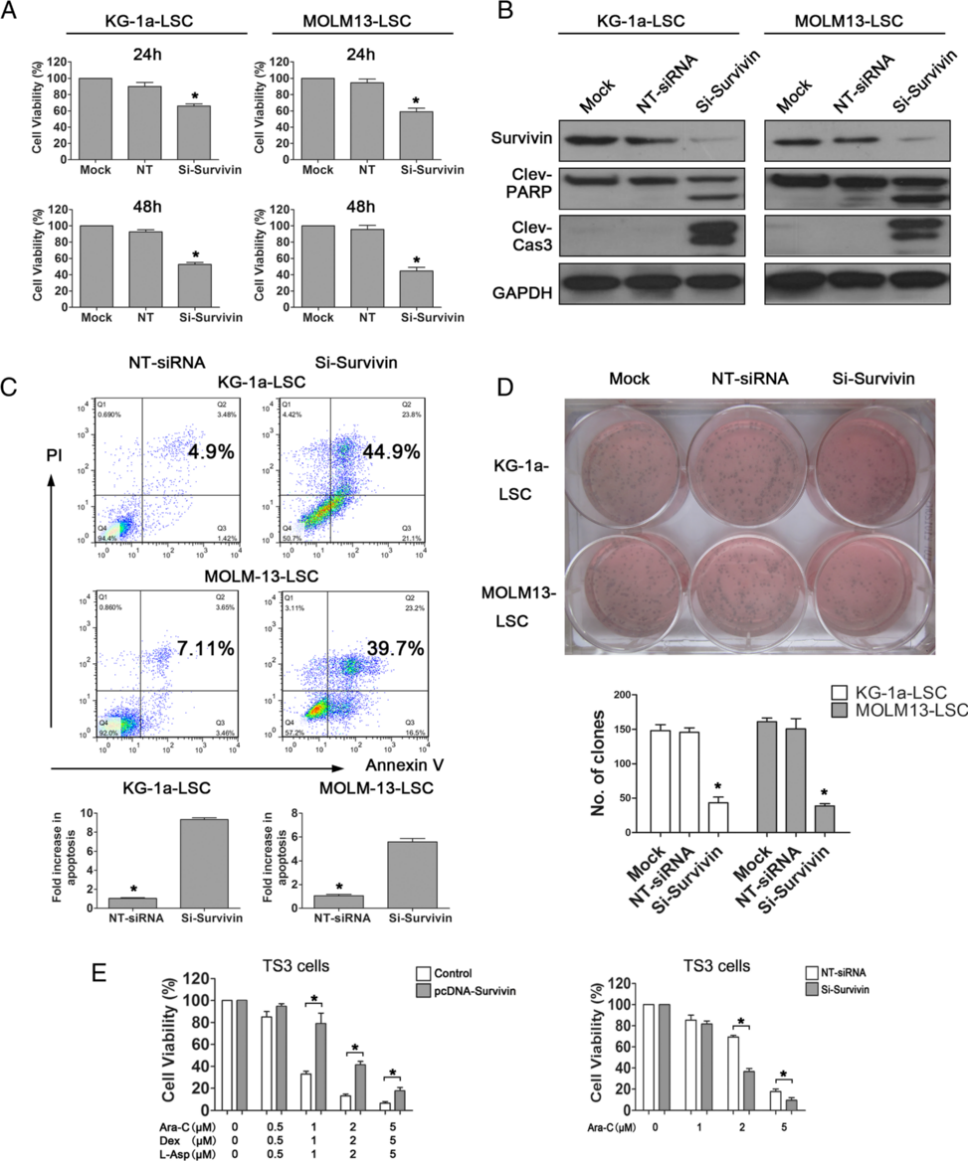
**Survivin knockdown abrogated LSCs growth, induced apoptosis, decreased self-renewal, and sensitized cells to chemotherapy. (A)** Analysis of LSCs viability 24 or 48 h after transfection with survivin-siRNA (^*^ *P* < 0.05). **(B)** Analysis of survivin protein expression, cleaved PARP, and caspase3 expression 48 h after transfection with survivin-siRNA in KG-1a and MOLM13LSCs. Non-targeting (NT)-siRNA was used as negative control. **(C)** Analysis of apoptosis rates in LSCs 48 h after transfection with survivin-siRNA (^*^ *P* < 0.05). NT-siRNA was used as a negative control. **(D)** Analysis of the self-renewal capacity of LSCs following knockdown of survivin (^*^ *P* < 0.05). NT-siRNA was used as a control. **(E)** Primary AML CD34+ cells (TS3 cells) were transfected with either pcDNA-survivin vectors or siRNA and treated with combined therapy (left panel) or Ara-C alone (right panel) at the indicated concentrations (^*^ *P* < 0.05). Cell viability was detected by CCK-8 assay.

Next, we conducted loss of survivin function to investigate its contribution to LSCs apoptosis-related characteristics. Specific siRNAs were used to knockdown survivin, and a non-targeting siRNA was used as control. CCK-8 assays showed significant decreases (*P* < 0.05) in LSCs growth at 24 and 48 h after transfection (Figure 2A). Similarly, marked survivin knockdown was observed, and cleaved PARP and caspase3 were obviously increased with survivin-siRNA 48 h after transfection in both LSC-enriched cell lines (Figure 2B). Meanwhile, annexin-V/PI FACS analysis confirmed the obvious increase in apoptosis at 48 h after siRNA transfection in LSCs (Figure 2C), suggesting that knockdown of survivin induced apoptosis in LSCs. Moreover, soft agar colony assays revealed that knockdown of survivin using survivin-siRNA significantly decreased colony numbers in KG-1a LSCs compared with the non-targeting control (43.3 ± 4.8 vs. 145.7 ± 3.2, respectively) and MOLM13 LSCs (38.6 ± 1.7 vs. 150.6 ± 2.3, respectively; *P* < 0.05, Figure 2D).

Subsequently, to investigate whether survivin expression promoted drug resistance, we analyzed drug resistance AML cell line TS3 (isolated from CD34+ cells), established from a patient who relapsed despite treatment with chemotherapy, and used for further experiments. Transduction of primary AML cells (TS3) with a pcDNA-survivin construct obviously attenuated the effects of Ara-C, Dexamethasone and L-Asp alone or in combination on cytotoxicity as compared with controls (*P* < 0.05), indicating that over-expression of survivin renders primary AML cells more resistance to chemotherapeutic agents (Figure 2E left panel). Conversely, to determine whether AML cells can be sensitized to chemotherapeutic drugs, we specifically silenced survivin with si-RNA in TS3 cells. Non-targeting controls yielded an approximately 2.5-folder higher IC_50_ (4.5 μM) than the survivin-siRNA group (IC_50_ = 1.8 μM, *P* < 0.05, Figure 2D right panel), demonstrating that down-regulation of survivin could overcome drug resistance in primary AML cells. Taken together, these results suggested that survivin contribute to the anti-apoptotic and drug-resistant characteristics of LSCs, thereby supporting further investigation of the potential mechanism of survivin over-expression in these phenotypes.

### Survivin promoter region is participated in regulation of transcription levels

**Figure 3:**
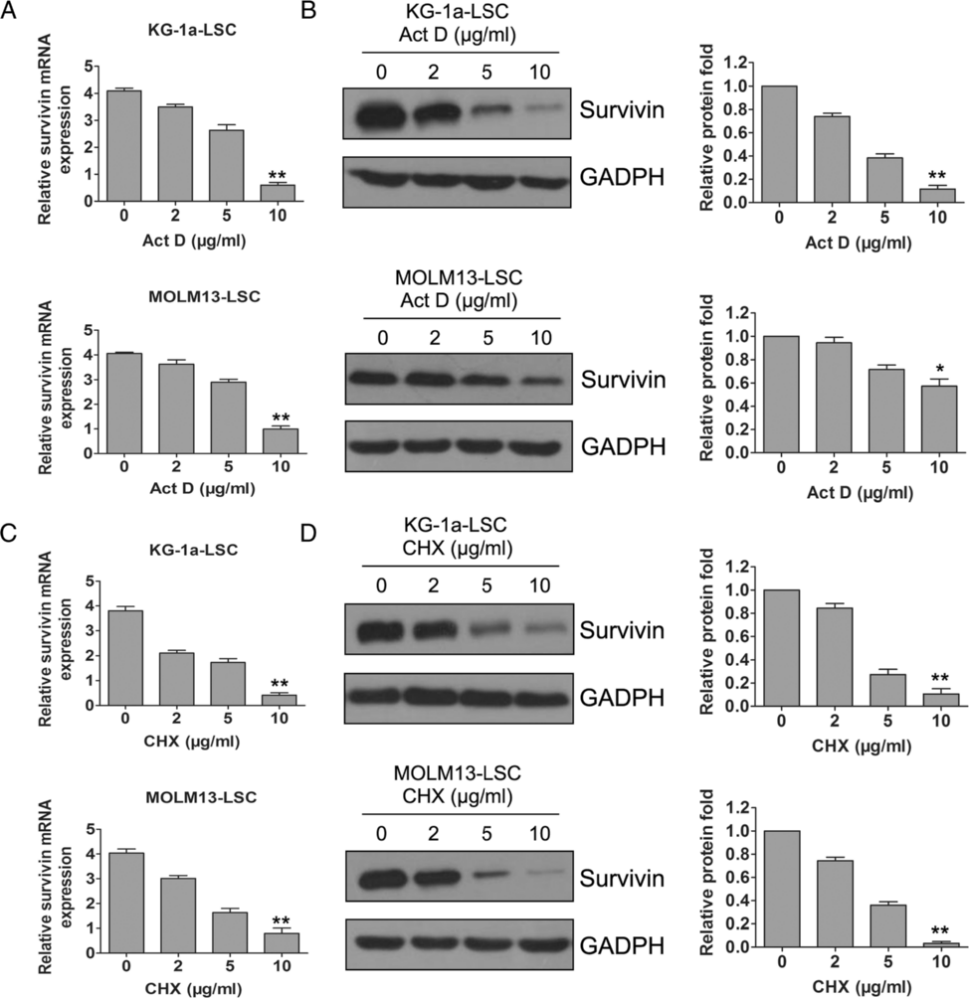
**Survivin expression was controlled by transcription and translation. (A-B)** Survivin mRNA and protein expression was determined after treatment with the transcriptional inhibitor actinomycin D (Act D) in boht LSCs cell lines. **(C-D)** Survivin mRNA and protein expression was determined after treatment with the translational inhibitor cycloheximide (CHX) in boht LSCs cell lines.

Survivin mRNA is extremely stable in numerous cancers, and the regulation of this oncogene in tumors can be linked to translational control [23,24]. Therefore, to determine whether survivin mRNA (and protein) was also stable in LSCs, we treated LSCs with the transcriptional blocker actinomycin Act or the translational inhibitor cycloheximide (CHX). Survivin mRNA and intracellular protein levels were largely dependent on regulation of constitutive transcription and translation, both of which levels were dramatically reduced after 24 h of treatment in both LSC cell lines (Figure 3).

**Figure 4:**
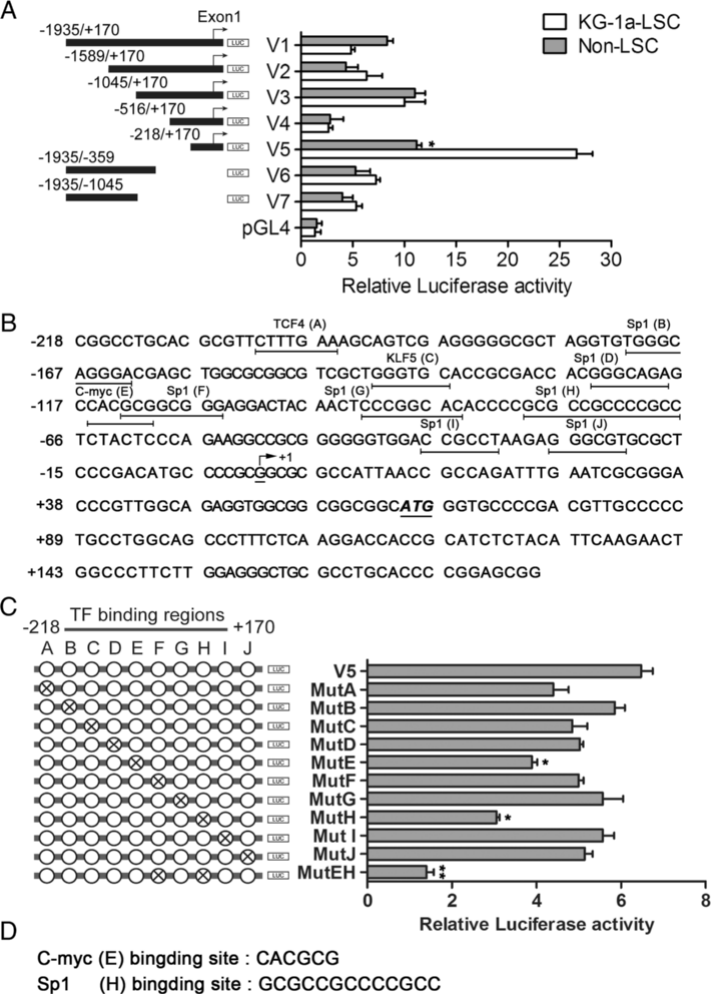
**Identification and characterization of survivin core promoter region. (A)** Relative luciferase activity of different survivin promoter constructs (5′-deletion and 3′-deletion) in KG-1a-LCSs and non-LSC subpopulations. The cells were transfected with 1 μg of promoter plasmid along with 50 ng of β-actin-*Renilla* luciferase plasmid as control. **(B)** The core promoter region of survivin was identified (-218 to +170), and several transcription factor binding sites were predicted using Mat-Inspector and Jaspar (underlined regions). The arrow denotes the start site of survivin transcription, and the initiation codon ATG is shown in bold and italicized font. **(C)** Mutation of each transcription factor binding site was performed for identification of potential trans-activation motifs. Luciferase assays showed changes in promoter region activity for the mutants versus wild-type construct (^*^ *P* < 0.05; ^**^ *P* < 0.01). **(D)** Mutant E and mutant H are c-Myc and Sp1 binding sites, respectively; the sequences of the two motifs are shown. All graphs represent at least triplicate independent experiments.

To further understand the transcriptional regulation of survivin in LSCs, we examined the activity of the promoter from the region ~ 2000 bp upstream of the transcription start site (TSS). Deletion constructs showed that promoter activity an increase stepwise (V2 and V3) or decreased slightly (in V4). Subsequent analysis of the luciferase activity of 3′-deletion constructs revealed similar results in V6 and V7. Interestingly, the V5 construct (-218 to +170; the shortest construct), exhibited 17.7- and 7.8-fold transcriptional activity in KG-1a-LSCs and Non-LSCs respectively, compared with the empty pGL4.7 vector control (Figure 4A).

The robust promoter activity obversed for the V5 sequences suggested a role for this core region in regulating survivin transcription. We next analyzed this region for transcription factors that could regulate survivin expression and identified several putative transcription factor-binding sites (Figure 4B). To verify which motifs mediated the transcriptional activity of the survivin promoter, substitution mutations of these individual sites (TCF4, KLF5, Sp1 and c-Myc) were prepared (primers for mutation are listed in Additional file 4: Table S2). Abrogation of individual sites repressed transcriptional activity by 30% and 50% in the MutE and MutH sites, respectively, and combined MutE/MutH decreased transcriptional activity by 85% compared with the wild-type promoter V5 sequence in KG-1a-LSCs. Moreover, none of the other mutant sites showed significant decrease in transcriptional activity as compared with the control (Figure 4C). Subsequently, we found that MutE and MutH represented the canonical E-box site (c-Myc sites) and tandem GC boxes (Sp1 sites) in the V5 promoter region (Figure 4D), indicating its vital function for transcription regulation.

### Transcription factor Sp1 and c-Myc activated survivin transcription in LSCs

**Figure 5:**
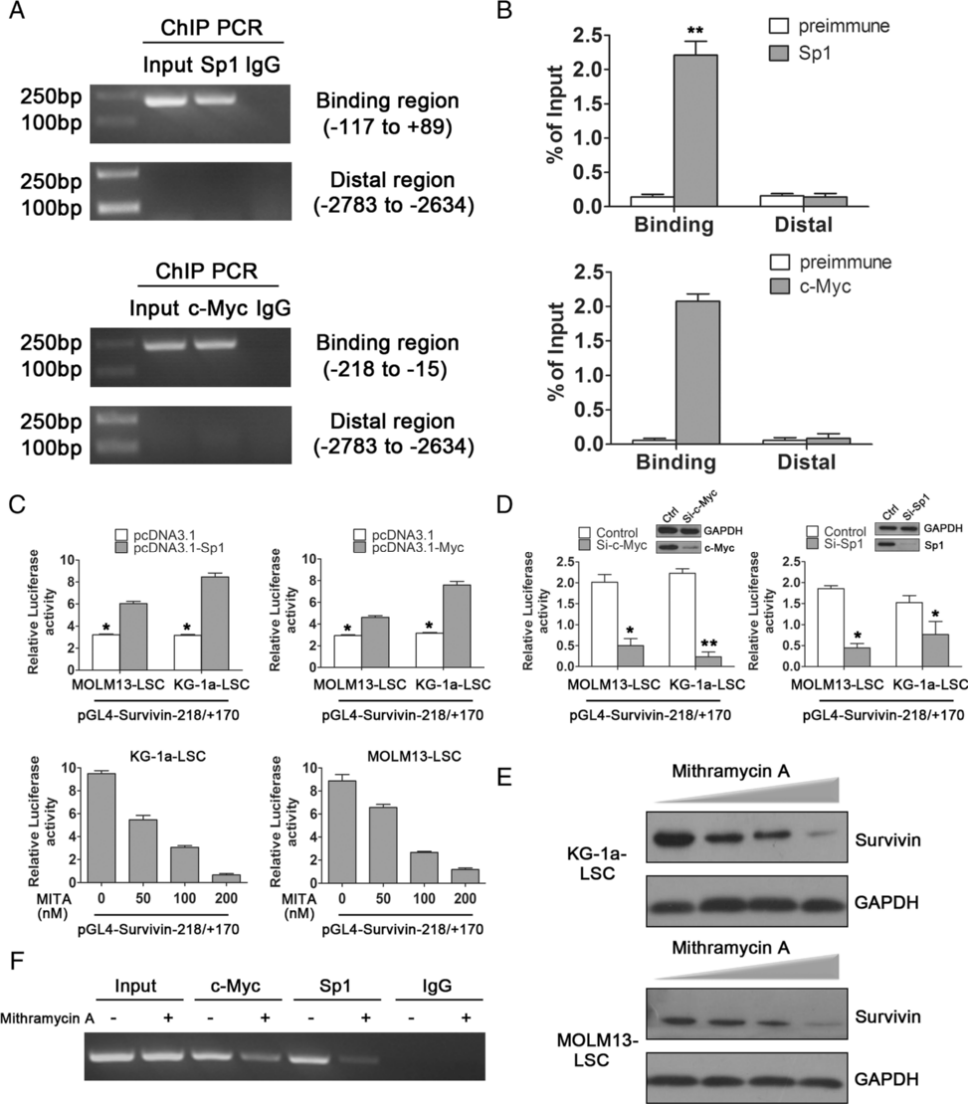
**Sp1 and c-Myc activated survivin transcription in LSCs. (A)** Chromatin immune-precipitation was used to analyze the binding of Sp1 and c-Myc to the survivin promoter. Input was used as a positive control. **(B)** ChIP-qPCR was used to confirm the results shown in **(A)** (^**^ *P* < 0.01). **(C)** Transcription activities of survivin was analyzed following over-expression of Sp1 and c-Myc vector in KG-1a and MOLM13-LSCs (^*^ *P* < 0.05). The cells were co-transfected with the pcDNA-survivin vector, core promoter V5, and *Renilla* luciferase plasmid as a control. Cells were also treated with MITA to analyze the effects of Sp1 inhibition on survivin promoter activity. **(D)** Specific siRNA-Sp1/c-Myc was performed to assess the effects of these two motifs on survivin luciferase activities. **(E)** Survivin protein levels were analyzed in LSCs following treatment with MITA at 50, 100, or 200 nM. **(F)** ChIP assays were used to determine whether MITA could directly inhibit Sp1 and c-Myc binding to its specific sites. All data are shown as the means ± SDs of results from three experiments.

To verify whether Sp1 and c-Myc could truly interact with the survivin promoter *in vivo*, we conducted chromatin immune-precipitation (ChIP) assays by incubating KG-1a-LSC cell nuclear extracts in the presence of anti-Sp1 and anti-c-Myc antibodies or IgG (as a negative control). ChIP primers for Sp1 (-117/+89) and c-Myc (-218/-15) were designed to amplify promoter regions containing putative binding sites; the distal region primers were used as a negative control. As shown in Figure 5A, ChIP with antibody against Sp1 and c-Myc cross-linked to the both binding site cluster, showed an enrichment of the survivin promoter compared with the IgG control in KG-1a-LSCs. The ChIP-qPCR also exhibited the similar results with the same primers (Figure 5B).

To further investigate the roles of Sp1 and c-Myc in the regulating of survivin promoter activity, ectopic over-expression of pcDNA-Sp1 and pcDNA-Myc co-transfected with the promoter construct V5 in KG-1a-LSCs and MOLM13-LSCs resulted in distinct elevation in luciferase activities compared to cells transfected with empty vector (Figure 5C and Additional file 3: Figure S3C). Conversely, we used mithramycin A (MITA), an inhibitor of Sp1 and c-Myc, which inhibits transcriptional activation by preventing Sp1 from binding to GC-rich regions and blocking c-Myc expression [25], to elucidate these two motifs at various concentrations for 48 h. Promoter activity was significantly attenuated in MITA-treated cells from both cell lines (Figure 5C, lower panel). Specific siRNA-Sp1/c-Myc was also confirmed the marked decrease in luciferase activities of V5 construct in both LSCs cell lines, non-target siRNA was used as control (Figure 5D). Consistent with this, survivin protein expression was suppressed after treatment with MITA in a concentration-dependent manner (Figure 5E). Moreover, ChIP assays demonstrated that MITA could repress Sp1 and c-Myc in transcription level (Figure 5F). Taken together, these results indicated that Sp1 and c-Myc could directly bind to the predicted sites in the survivin core promoter region V5 and were crucial for the transcriptional activation of survivin expression.

### c-Myc required functional Sp1 binding sites for transcriptional regulation of the survivin promoter

**Figure 6:**
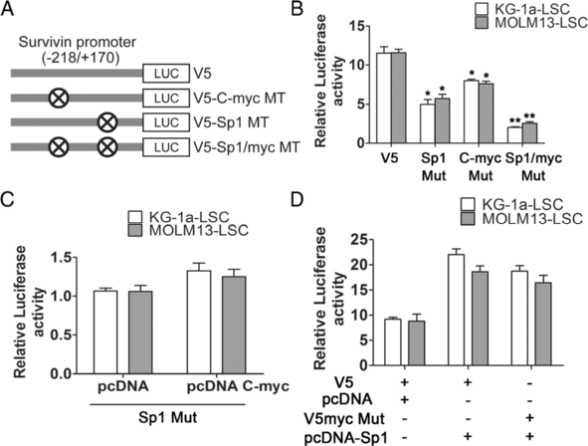
**c-Myc required functional Sp1 binding sites to activate survivin transcription. (A)** c-Myc and Sp1 mutant constructs used for this study. **(B)** The effects of mutation of Sp1, c-Myc,and combined mutation of the two motifs on the relative luciferase activity of the wild-type V5 promoter in KG-1a and MOLM13LSCs. The mutant and wild-type constructs were transfected along with *Renilla* luciferase plasmid as control (^*^ *P* < 0.05, ^**^ *P* < 0.01). **(C)** The effects of c-Myc over-expression on survivin promoter activity in the context of mutated Sp1 binding sites. The mutant Sp1 site constructs were co-transfected with pcDNA-c-Myc or empty vector and *Renilla* luciferase plasmid. Luciferase assays were performed 48 h post-transfection. **(D)** The effects of Sp1 over-expression on survivin promoter activity in the context of mutated c-Myc binding sites. All transfection conditions were performed as previous described.

To comprehensively identify whether these two factors contributed equally or display different degrees of trans-activation of the survivin promoter, point mutations were created in both Sp1 and c-Myc, and mutant constructs were transfected alone or in combination into KG-1a and MOLM13-LSCs (Figure 6A). As shown in Figure 6B, we observed ~50% and ~40% decreases in promoter activity in the elements carrying a mutant GC-box region (Sp1) or E-box region (c-Myc), and combination mutants result in a nearly 80% decrease in luciferase activity, as compared with the wild-type promoter V5 -218/+170 in both cell lines. Next, we further investigated the interplay between Sp1 and c-Myc in survivin promoter. When c-Myc was over-expressed in the presence of a mutated Sp1 binding site, evaluation of luciferase activity revealed that the V5 promoter was not significantly activated as compared with the empty pcDNA vector control (Figure 6C). These data indicated that mutation of the Sp1 binding site affected the transcriptional activation of c-Myc. In contrast, Sp1 over-expression was significantly increased by ~2-fold in the presence of mutated c-Myc sites (*P* < 0.05), and the increased activation was similar with that of the wild-type promoter relative to the empty pcDNA vector control (Figure 6D). Thus, mutation of the c-Myc binding site did not affect transcriptional activation by Sp1. In general, these results revealed that Sp1 possessed the predominantly role in transcriptional regulation of the survivin promoter, and that the downstream function of c-Myc mainly depended on the integrity of Sp1 binding sites.

### Inhibiting Sp1 and c-Myc-dependent survivin expression caused chemo-sensitization

To determine whether inhibit of Sp1 or c-Myc could sensitize LSCs to anticancer drug-induced cyto-toxicity, we incubated KG-1a and MOLM13-LSCs with MITA in the presence of Ara-C (5 μM) for 3 days and measured the percentage of apoptotic cells using flow cytometry analysis. As shown in Additional file 5: Figure S4A and S4B, MITA substantially increased the Ara-C induced apoptosis from 49.7% to 76.3% in KG-1a-LSCs and 46.4% to 63.6% in MOLM13-LSCs. Intriguingly, MITA also substantially reduced the tumor-sphere-forming ability in the presence of Ara-C in LSCs compared with the MITA only and Ara-C only groups (*P* < 0.01, Additional file 5: Figure S4C). In other words, MITA combined with chemotherapeutic drugs could effectively reduce the self-renewal of LSCs, which represent for tumor unlimited proliferation and recurrence. Consistent with this, MITA distinctly repressed luciferase activity in the presence of Ara-C compared with DMSO in both cell lines (*P* < 0.05, Additional file 5: Figure S4D). Collectively, these results indicated that inhibiting Sp1 and c-Myc-dependent survivin expression could cause chemo-sensitization in LSCs, suggesting that survivin could be an effective target for further clinical applications for AML treatment.

### High expression of Sp1 and c-Myc in CD34+ AML samples correlated with Survivin expression

**Figure 7:**
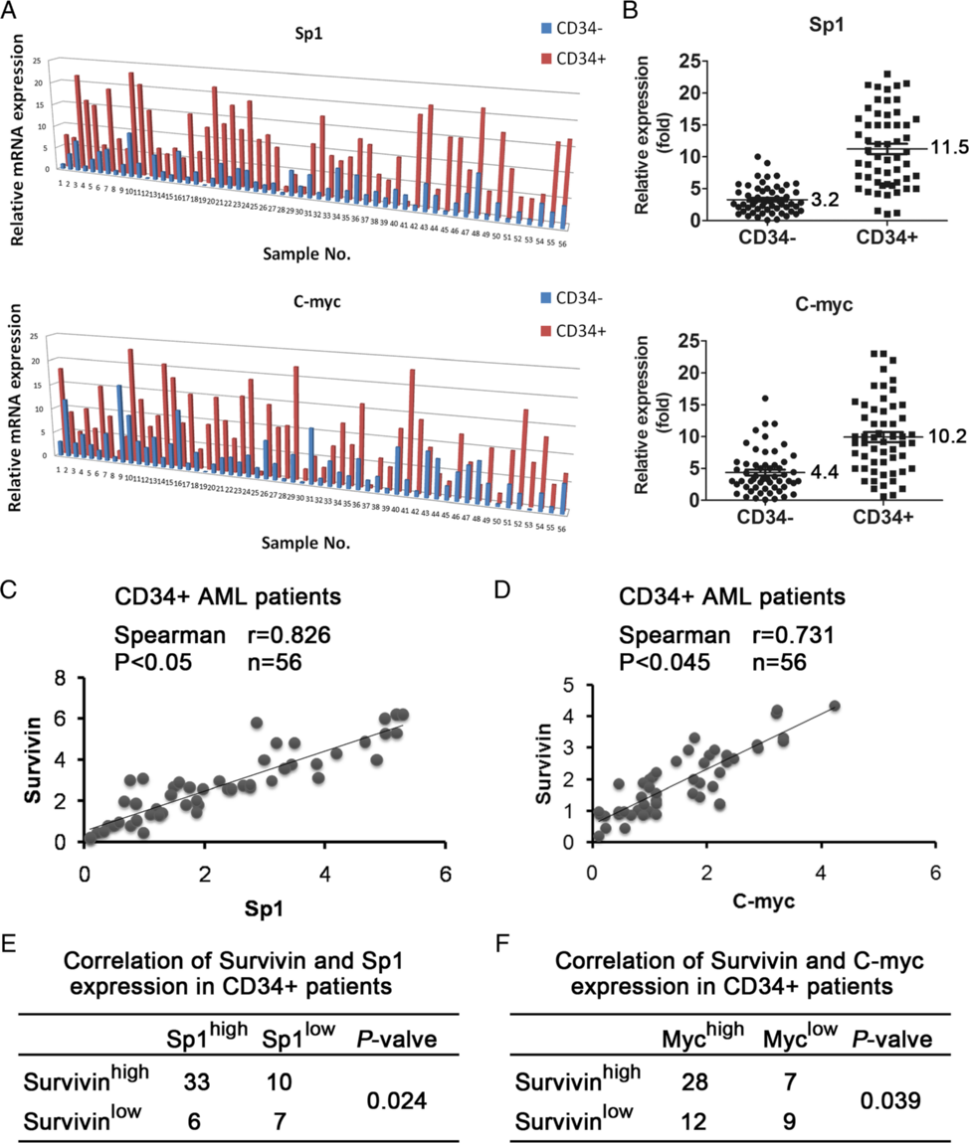
**Expression of Sp1 and c-Myc in CD34+ AML patients and correlations with survivin levels. (A)** A total of 56 paired CD34+ and CD34- AML patient samples were collected. For each sample, the expression levels of Sp1 and c-Myc were determined by RT-qPCR and normalized to GAPDH as an internal control. **(B)** The relative expression of each sample is illustrated. The scatter dot plot shows the relative levels of Sp1 and c-Myc in CD34+ and CD34- cells from AML patients. **(C-D)** Correlations among survivin, Sp1, and c-Myc mRNA levels in CD34+ AML patients, as determined using RT-qPCR and Spearman’s analysis. **(E-F)** Correlations among survivin, Sp1, and c-Myc protein levels CD34+ AML patients (n = 56), as determined using Fisher’s exact probability test.

To understand the clinical significance of Sp1 and c-Myc, paired CD34+ and CD34- cells from 56 AML patients were analyzed, and relative mRNA levels were determined as shown in Figure 7A. Both Sp1 and c-Myc were substantially up-regulated in CD34+ AML samples compared with their differentiated counterparts. Approximately 80% (45/56) and 64% (36/56) of the CD34+ cells displayed more than 2.2-fold higher expression of Sp1 and c-Myc than that by the CD34- cells. On average, Sp1 and c-Myc levels were elevated by 3.5- and 2.3-fold, respectively, in CD34+ AML samples (Figure 7B).

On the basis of the above observations, we next investigated whether Sp1 and c-Myc were expressed consistently with survivin in CD34+ AML patient samples. Linear regression analysis demonstrated a positive correlation between the mRNA levels of both survivin and Sp1 (*P* < 0.05, r = 0.826) and c-Myc (*P* < 0.035, r = 0.731) in CD34+ AML samples (Figure 7C and D). We then determined the protein levels of Sp1, c-Myc and survivin in 56 AML patients by immuno-blotting (quantification by Quality One software, high expression was calculated as more than 2 folder compared with CD34- parts, and low expression was calculated as less than 0.5 folder, data not shown). As shown in Figure 7E and F, there were 33 and 28 specimens that showed high expression of both Sp1/c-Myc and survivin, and 7 and 9 specimens that showed low expression of both Sp1/c-Myc and survivin. Evaluation these datas with Fisher’s exact test revealed that significant correlations between survivin protein levels and Sp1/c-Myc protein levels in CD34+ AML specimens (*P* = 0.024 and *P* = 0.039, respectively). Collectively, these results suggested that Sp1 and c-Myc over-expression in CD34+ AML samples regulated survivin transcription, and that both the mRNA and protein levels of Sp1 and c-Myc were positively correlated with survivin expression.

### The ERK/MSK MAPK pathway was constitutively active in primary CD34+ AML samples and was required for Sp1 expression

**Figure 8:**
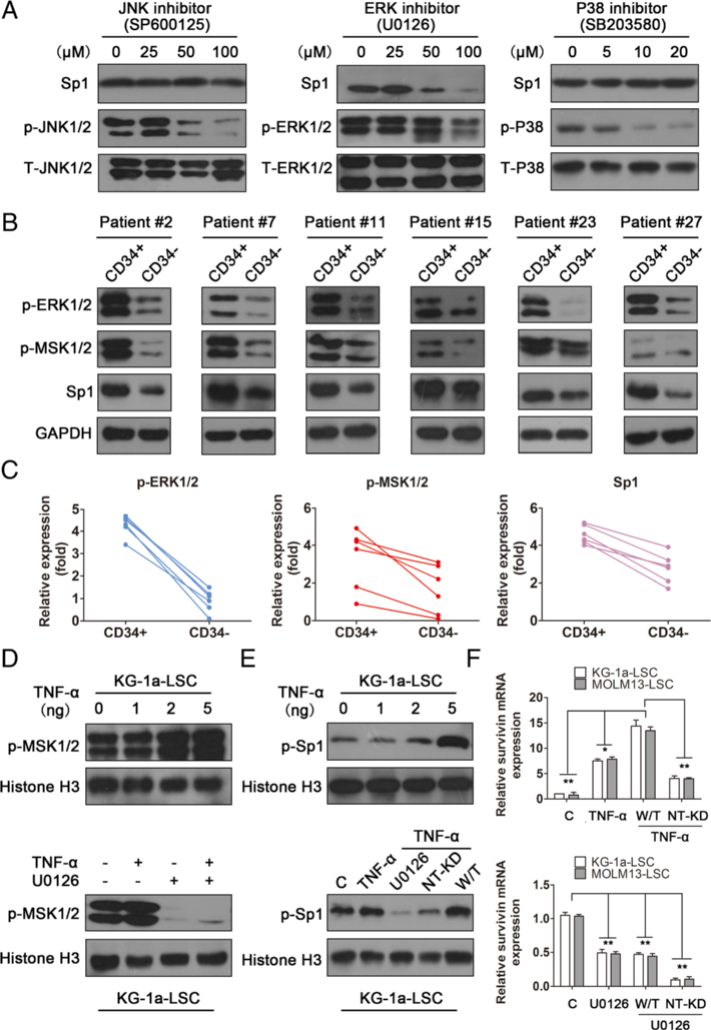
**Constitutive activation of the ERK/MSK/Sp1 axis was required for survivin expression in CD34+ AML samples. (A)** LSCs were treated with the JNK inhibitor SP600125 (0, 25, 50, or 100 μM), the ERK inhibitor U0126 (0, 25, 50, or 100 μM), or the p38 inhibitor SB203580 (0, 5, 10, or 20 μM) for 48 h, and survivin protein expression was analyzed by immune-blotting. **(B)** The phosphorylation levels of ERK and MSK, as well as the protein expression level of Sp1, were analyzed in six paired CD34+ AML samples by immune-blotting, and GAPDH was used as an internal control. **(C)** The density of each protein band was quantified, and the lines indicate the tendency of altered levels of p-ERK, p-MSK, and Sp1 in each paired sample. **(D)** Analysis of MSK activation following treatment with TNF-α or the ERK inhibitor U0126. **(E)** Analysis of Sp1 activation following stimulation with TNF-α or the ERK inhibitor U0126. Results were confirmed using the NT-KD MSK vector and the WT MSK vector. **(F)** Analysis of survivin mRNA following treatment with TNF-α, U0126, or NT-KD MSK in LSCs (^*^ *P* < 0.05, ^**^ *P* < 0.01).

Because survivin transcription was mainly regulated by Sp1, we next examined pathways that regulate Sp1 in LSCs. Various signaling pathways, including the MAPKs, have been linked to the expression of Sp1 in multiple cancers. Therefore, inhibitors of the ERK (U0126), JNK (SP600125), and p38 (SB203580) pathways were used to characterize the potential role of MAPKs in the constitutive expression of Sp1. As shown in Figure 8A, only the ERK inhibitor (U0126) markedly reduced Sp1 protein expression, whereas JNK and p38 inhibitors did not significantly affect Sp1 protein levels. To determine which molecules were involved in signaling downstream of the ERK MAPK pathway to modulate Sp1 gene expression, the phosphorylation of MSK, which can be activated by MAPK/ERK and p38, was analyzed in six paired CD34+ and CD34- AML patients. As expected, phosphorylated-ERK, phosphorylated-MSK, and Sp1 were obviously constitutively active in all CD34+ fractions (Figure 8B) and were upregulated by 4.8-, 2.0-, and 1.7-fold on average, respectively (Figure 8C). To investigate whether ERK activated phosphor-MSK, we used tumor necrosis factor (TNF)-α, a specific activator of ERK, and U0126, a specific inhibitor of ERK. The result showed that MSK phosphorylation was elevated after TNF-α stimulation in a concentration-dependent manner (Figure 8D upper panel). In contrast, pretreatment with 20 μM U0126 markedly inhibited TNF-α-induced MSK phosphorylation (Figure 8D lower panel), indicating that MSK was regulated by ERK.

**Figure 9:**
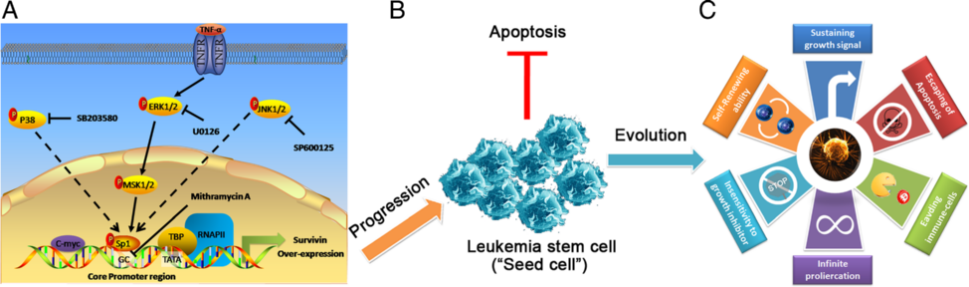
**The proposed mechanism for survivin overexpression in LSCs. (A)** TNF-α stimulation leads to transactivation of TNFR, which activates ERK/MSK/Sp1 pathways. This activation, together with c-Myc, concurrently regulates survivin expression in LSCs, facilitating LSCs growth, allowing LSCs escape from apoptosis **(B)**, and finally permitting the evolution to the “Seed cell” in acute myeloid leukemia **(C)**. The straight arrow represented activation, the dotted arrow represented none affection, and TBP and RNA PII represented the transcription initiation complex.

Furthermore, because MSK mediates cytokine-induced Sp1 phosphorylation at Thr^453^ [26], we next examined whether TNF-α could induce the phosphorylation of endogenous Sp1 via the ERK/MSK pathway using a phospho-specific Sp1 Thr^453^ antibody. Substantial phosphorylation of Sp1 was observed upon stimulation with TNF-α (Figure 8E upper panel). Subsequently, we constructed a vector-encoding mutant MSK (NT-KD), in which the N-terminal kinase domain was inactivating by a point mutation, and a vector encoding wild-type MSK. Pretreatment with U0126 or NT-KD MSK markedly inhibited the phosphorylation of Sp1 as compared with the WT MSK vector,and histone H3 was used as control (Figure 8E lower panel). Additionally, the survivin mRNA level was significantly up-regulated by TNF-α and W/T MSK vector when compared with NT-KD (*P* < 0.01, Figure 8F upper panel). Conversely, transfections of W/T MSK vector could partly alleviate the decreased mRNA level caused by U0126 in both cell lines 48 h after treatment (*P* < 0.05, Figure 8F lower panel). Taken together, our data supported that constitutive activation of the ERK/MSK/Sp1 axis (as shown by phosphorylation of the pathway components) was required for over-expression of survivin in CD34+ AML patients, and finally permitting the evolution to the “Seed cell” in acute myeloid leukemia (Figure 9).

## Discussion

AML is typically a stem cell-related disease, and the existence of LSCs was the first identified in tumor-initiating cells. Strategies to target such cells, which have been shown to be resistant to conventional treatments, are expected to greatly improve clinical outcomes and overall survival rates [27]. Our data presented herein demonstrated that survivin was highly expressed specifically in LSC-enriched fractions and in approximately 85% of CD34+ AML patients compared to their differentiated counterparts, thereby contributing to drug resistance. However, the over-expression of survivin was not associated with clinical characteristics, such as age, sex, FAB subtype, or laboratory parameters (Table 1), indicating that survivin could be an independent factor for diagnosis or prognosis of AML. Moreover, the expression of survivin was transcriptionally up-regulated by Sp1 and c-Myc via the ERK-MSK pathway, and constitutively active (phosphorylated) ERK/MSK in CD34+ AML patients was required for the phosphorylation (i.e., activation) of Sp1, thereby leading to over-expression of survivin. Thus, we showed for the first time that Sp1/c-Myc enhanced survivin expression in LSCs via an ERK/MSK/Sp1/c-Myc-dependent mechanism, maintaining and promoting chemo-resistance.

Numerous studies, including clinical trials of solid tumors, acute lymphoblastic leukemia (ALL), and large B-cell lymphoma, have shown that survivin is an important target in various cancers. Nakahara T *et al*. [28] proposed that targeting survivin with the novel small-molecule inhibitor YM155 could obviously suppress the growth of primary prostate tumor xenografts *in vivo*. Additionally, Kwee J. K *et al*. [29] reported that survivin could induces mitochondrial fragmentation, and reduces mitochondrial respiration, thereby preventing the accumulation of reactive oxygen species, inhibiting apoptosis, and promoting drug resistance. Consistent with these reports, our results also indicated that inhibition of survivin could dramatically decrease LSCs growth, induces apoptosis, suppress self-renewal, and sensitizes cells to chemotherapy. However, the regulation of survivin expression in CSCs is poorly understood. Thus, a better understanding of the regulation of survivin in LSCs is required for the development of strategies to target these cells. Our results found that steady-state expression of survivin required constitutive transcription and translation in LSCs; therefore, we focused on elucidating the mechanism of transcriptional activation in LSCs.

In this study, the *cis*-acting promoter elements and respective binding factors required for constitutive activity of the survivin promoter in LSCs were identified and characterized. Deletion analysis of the survivin promoter in LSCs revealed a core region from nucleotide -218 to nucleotide +170, and Sp1/c-Myc played crucial roles as potent *cis*-acting elements to regulate survivin transcription during carcinogenesis. Moreover, Sp1 and c-Myc directly interacted with the core promoter region (-218 to +170) *in vivo*, and over-expression or knockdown of either Sp1 and c-Myc significantly enhanced or attenuated activity of the survivin core promoter (V5) and endogenous survivin expression, indicating that the high expression of survivin in LSCs was dependent on the trans-activation of Sp1 and c-Myc through positioning of these transcription factors at specific sequence sites.

Sp1, a C_2_H_2_-type zinc finger-type transcription factor that binds GC-rich sequences, was one of the first eukaryotic transcription factors to be identified and has been shown to play an important role in the transcriptional regulation of various genes. Its importance is highlighted by the observation that Sp1^-/-^knockout mice exhibit embryonic lethality [30]. Sp1 is ubiquitously expressed in normal tissues and elevated in tumors, correlating with a wide range of cellular events and different cell-type roles in transcription procession [31,32]. Here we found that the constitutive activation of ERK and MSK was required for Sp1 expression, and phosphorylation Sp1 was regulated by TNF-α and ERK/MSK signaling, which ultimately activation survivin expression with c-Myc in LSCs cell lines and clinical specimen. ERK pathway activation has been shown to play a role in the stability of transcription factors [33-35], which may also be the basis for the effects of ERK inhibition on SP1 expression seen in this study. Additionally, our results also showed that Sp1 expression was significantly up-regulated in 92% of paired CD34+ AML patients and correlated with the expression of survivin mRNA and protein, indicated that Sp1 was a specific and critical factor mediating survivin expression.

c-Myc is another pivotal trans-activation factor for the survivin promoter, as identified in our study. Myc protein belongs to the Max of Bhlh-Zip family, the members of which bind to E-boxes and regulate transcription of target genes as obligate heterodimers with a parter protein [36]. Myc functions as a proto-oncogene and is frequently up-regulated in a wide range of tumor types, driving proliferation and tumor progression. Myc is also known to mediate the reprogramming of somatic cells [37] and participates in regulation of the cell cycle and stabilization of cell division [38]. In the context of gene regulation, Myc acts as a weak transcription factor, facilitating the formation of transcription complexes [39]. In the present study, we demonstrated that Myc over-expression promoted activation of the survivin promoter in LSCs; however, this effect was only marginal when Sp1 binding sites were mutated, suggesting that Myc requires functional Sp1 sites to regulate survivin expression. Similarly, c-Myc expression was significantly up-regulated in 91% of paired CD34+ AML patients and correlated with the expression of survivin mRNA and protein. Taken together, our data suggested that while Sp1 functioned independently to regulate the survivin promoter, c-Myc required Sp1 function in order to participate in the regulation of survivin. Further studies are required to determine whether this cooperation between Sp1 and Myc also functions to regulate the expression of other oncogenic genes in cancer.

**Figure 10:**
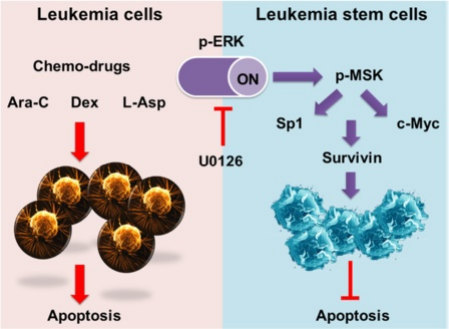
**Phosphorylation ERK was specific activated in LSCs.** Phosphorylation ERK was intrinsic and spontaneous activated in LSCs, which led to drug resistance for clinical chemo-agents. In contrast, the bulk leukemia cells were more sensitive to chemical drugs and eventually involved in apoptosis.

The MAPK signal pathway (ERK, JNK, p38) is involved in regulating cell growth, differentiation, environmental adaptation to stress, inflammatory reactions, and participated in cancer progression [40]. In this study, we showed that only the ERK MAPK pathway played a role in the regulation of Sp1 levels in LSCs, inhibition of JNK or p38 did not affect Sp1 expression. Importantly, ERK and its downstream signaling mediator MSK were obviously constitutively activated (i.e phosphorylated) in six paired CD34+ AML patients, suggesting that activation of ERK and MSK (as observed by phosphorylation levels of these targets) was intrinsic and spontaneous in such cells and was required for the expression of survivin in LSCs (Figure 10). MSK is known to be regulated by the MAPK/ERK and SAPK2a/p38 pathways and is currently the best candidate for the regulation of cytokine-induced phosphorylation of transcription factors [41-43]. Interestingly, we found that MSK activated Sp1 by phosphorylation at Thr^453^. Collectively, our results showed that Sp1 activation was involved in the signaling pathway downstream of ERK and MSK, mediating TNF-α-induced expression of survivin.

From our results showing the correlations between Sp1 and c-Myc levels and survivin expression in CD34+ AML patients, inhibitors of Sp1 and c-Myc may provide novel therapeutic strategies for the treatment of leukemia. MITA, an anticancer antibiotic that selectively binds to G-C-rich DNA domains in the presence of Mg^2+^ or Zn^2+^, inhibits Sp1 transcriptional activity by preventing its binding into the DNA major grooves. We found that MITA treatment effectively suppressed survivin promoter activity and reduced survivin mRNA and protein levels in LSCs. Moreover, MITA could sensitize LSCs to chemotherapy (such as Arc-C) and decreased the self-renewal ability of LSCs, suggesting that MITA may have potential applications in the treatment of leukemia (Additional file 5: Figure S4). However, there are also some limits in our study. First, we should expand the sample sizes to further elucidate the relationship between survivin, Sp1 and c-Myc. Second, we only collected blood samples from AML for these experiments, and bone marrow samples from AML should also adapt in further investigation for the subsequent research. Additionally, we observed significantly decreased survivin expression and reduced Sp1-DNA binding activity after treatment with TNF receptor signaling inhibitors in our preliminary studies (data not shown). Therefore, the TNFR/ERK/MSK/Sp1/c-Myc pathway is likely involved in survivin over-expression in LSCs. Future studies will focus on the upstream signaling cascades that alternatively contribute to survivin over-expression.

## Conclusion

Our findings may provide new insights into the transcriptional regulation of survivin, and described the chemo-resistant properties of LSCs, which will facilitate the development of novel therapy strategies or identification of selective biomarker for diagnosis. Further investigations are warranted to definitively confirm survivin as a marker for AML.

## Materials and Methods

### Cell isolation, culture, and transfection

The human leukemia cell lines K562, KG-1a, HL-60, MOLM13, ML-1, and U937 were cultured in RPMI-1640 medium supplemented with 10% fetal bovine serum and 1% penicillin/streptomycin. KG-1a and MOLM13 cells were separated and enriched for CD34 + CD38- cells using magnetic microbeads (Miltenyi Biotec, Auburn, CA, USA) and labeled with CD34-PE, CD38-FITC, or isotype control antibodies. Cells were analyzed and sorted on a Magnetic Sorter unit, and the purity and viability of isolated cells were routinely greater than 95%. The siRNA primer sequences are listed in Additional file 3: Figure S3B and were custom synthesized by Shanghai Sangon (Shanghai, China). After transfection, the inhibition efficiency was examined by western-blotting. Transfections were performed with Lipofectamine TM2000 according to the manufacturer’s protocol (Invitrogen Co. Carlsbad, CA, USA). All signal pathway inhibitors or activators were purchased from Selleck Chemicals.

### Patient collection and sample preparation

Peripheral blood (PB) samples were collected from 56 newly diagnosed AML patients after obtaining informed consent according to the procedures approved by the Human Experimentation Committee at the Overseas Chinese Hosptial of Jinan University and Sun Yat-Sen University Cancer Hospital (Guangzhou, China). The characteristics of these patients are summarized in Table 1. Samples were enriched for leukemia cells by using Ficoll density gradient centrifugation to obtain the mononuclear fraction and when then cryopreserved in freezing medium consisting of Cryostor CS10 (BioLife Solutions). Subsequently, CD34+/CD34- cells were separated from the leukemia-enriched portion by magnetic-activated cell sorting (MACS; Miltenyi Biotech) after incubation with anti-CD34 antibodies and IgG controls (BD Biosciences). The CD34+/CD34- cells were supplemented with B27 (1:50; Life Technologies, Carlsbad, CA, USA), 10 ng/mL basic fibroblast growth factor (bFGF) and 20 ng/mL epidermal growth factor (EGF) in DMEM/F12 media. All cells were incubated at 37°C in a humidified chamber with 5% CO_2_.

### Reverse phase protein array

KG-1a cell subsets (CD34 + CD38+, CD34 + CD38-, CD34-CD38+, and CD34-CD38-) were separated and normalized to a concentration of 1 × 10^4^ cells/μL, and whole-cell lysates were prepared. The detailed description and procedures of the protein array (RayBio Human Apoptosis Antibody Array G Series) methodology, including antibody and detection steps according to the manufacturer’s protocol. The slides were incubated for 2 h with validated primary antibodies in concentrations ranging from 1:250 to 1:1000. Subsequently, the secondary antibodies were used to amplify the signal for another 2 h, followed by precipitation of the stable dye. Positive and negative controls were positioned across the slide at the edge of each sample, as described below. Finally, the protein expression intensity level of each spot was measured using MicroVigene software (Vigene Tech, North Billerica, MA, USA). All signal numbers were used for data processing and calculation after standardization and topographic normalization, and analyses were performed using the R Statistical Programming Environment, version 2.4.2.

### Quantitative RT-PCR (qRT-PCR) analysis

Total RNA was extracted using TRIzol reagent (Invitrogen) and reverse transcribed into cDNA. The mRNA level was evaluated by RT-qPCR with SsoFast Eva-Green Supermix (Bio-Ras, Hercules, CA, USA) and was analyzed with a C1000Thermal Cycler (CFX96 Real-Time System, Bio-Rad). Relative expressionwas normalized to GAPDHas an internal control. The following PCR conditions were used on the LightCycler: 95°C for 5 s, 58°C for 5 s, followed by 40 cycles of 95°C for 15 s and 60°C for 1 min in a 10-μL reaction volume. The primer sequences for RT-qPCR arelisted in Additional file 4: Table S3.

### Western blot analysis

Cells were harvested, rinsed twice in ice-cold PBS, and kept on ice for 30 min in cell lysis buffer containing 1 mM PMSF with constant agitation. Insoluble cell debris was discarded following centrifugation for 10 min at 12,000 rpm at 4°C. The protein samples were separated by sodium dodecyl sulfate polyacrylamide gel electrophoresis (SDS-PAGE) on 10% gels and subsequently transferred to polyvinylidene (PVDF) membranes (Millipore). Immunoblotting was performed for survivin, Sp1, c-Myc, c-Jun N-terminal kinase (JNK), ERK, MSK, and p38, with GAPDH expression as an internal control.

### CCK-8 assay

Approximately 5.0 × 10^3^ cells were seeded into each well of a 96-well plate. The cells were then exposed to various concentrations of chemical agents or si-RNA for 24 or 48 h. After incubation, 10 μL CCK-8 was added to each well and cells were further incubated for 4 h. Cell viability was evaluated based on absorbance at 490 nm in a microplate absorbance reader (Bio-Rad).

### Flow cytometry and Annexin V-FITC/PI staining

CD34 and CD38 expression were assayed by flow cytometry (FACS Calibur, BD Company) using anti-CD34-PE and anti-CD38-FITC antibodies (BD Company). For apoptosis assays, cells were harvested at 48 h after drug treatment or transfection and centrifuged at 1500 × *g* for 5 min. After addition of 10 μL Binding reagent and 2.5 μL Annexin V-FITC (KeyGEN BioTECH), samples were suspended in 0.5 mL cold 1× Binding Buffer twice and stained with 10 μL PI. The percentage distributions of apoptotic cells were calculated by FlowJo software.

### Plasmids constructs, transient transfection, and luciferase assay

The whole promoter region of the human *surviving* gene was cloned into the pGL4 plasmid (Promega, Madison, WI, USA). To generate various 5′-deletion and 3′-deletion constructs of the surviving promoter, sequences were amplified from genomic DNA isolated from LSCs (Primers for deletion constructs are listed in Additional file 4: Table S1). A site-directed mutagenesis kit (Stratagene, Santa Clara, CA, USA) was used to generate transcription factor binding site mutant constructs in regions -218 to +170 of the survivin promoter. LSCs were cotransfected with 20 μg of the reporter plasmid and 1 μg *Renilla* luciferase control vector (Promega) as an internal control for normalization of transfection efficiency. The cells were then harvested at 24 h after transfection using a Dual-Luciferase reporter assay system (Promega), according to the manufacturer’s instructions. The whole gene lengths of Sp1,c-Myc, WT MSK, and NT-KD MSK were cloned into the pcDNA3.1 vector. Data are presented as the mean ratio for triplicate experiments.

### Sphere formation assay

Single LSCs were isolated and cultured for 2 weeks in methyl-cellulose medium (Stem Cell Technologies) supplemented with 20 ng/mL EGF (Sigma-Aldrich, St. Louis, MO, USA), 10 ng/mL basic fibroblast growth factor (bFGF; Invitrogen), 4 μg/mL insulin (Sigma-Aldrich), and B27 (1:50, Invitrogen). After treatment with chemical drugs, the tumor-sphere numbers were counted, and further statistical analyses were performed. All cell culture was carried out at 37°C in a humidified incubator with 5% CO_2_.

### Chromatin immune-precipitation (ChIP) assay

Confluent LSCs were cross-linked with 1% formaldehyde for 15 min at room temperature. The cross-linking reaction was terminated by the adding of glycine at a final concentration of 0.125 M. Subsequently, the lysed cells were isolated and sonicated on ice to shear DNA into fragments of 200 bp to 1 kb. Chromatin complexes were collected using EZ-Chip Chromatin Immuno-precipitation kit (Millipore) according to the manufacturers’ instructions. The chromatin was immune-precipitated for 16 h at 4°C using anti-Sp1, anti-c-Myc antibodies, and normal IgG (Millipore) as indicated. The input DNA was isolated from the sonicated lysates before immuno-precipitation as a positive control. The immune complexes were collected with Protein A/G Plus agarose (Pierce, Rockland, IL, USA), and the cross-links were then reversed by heating the samples at 65°C for 4 h. Purified DNA was then treated with proteinase K at 42°C for 1 h, and ChIP-PCR was performed as described.

### Statistical analysis

Statistical analyses were performed with SPSS version 13.0 (SPSS, Chicago, USA). Quantitative results are presented as the mean ± standard deviation (SD), and statistical analyses were carried out by t-tests. The Pearson χ^2^ test was performed to compare the frequency of survivin overexpression between paired AML patients with CD34+ and CD34- cell subsets. Associations between the protein level and continuous variables were assessed using Pearson’s and Spearman’s correlation and linear regression analyses. Correlations between the levels of Sp1, c-Myc, and survivin were assessed with Spearman’s analysis. Differences with *P* values of less than 0.05 were considered significant (^*^P < 0.05 and ^**^P < 0.01).

## Additional files

Additional file 1: Figure S1. Isolation and Identification of LSCs. (A) Six leukemia cell lines were selected and identified with the surface marker of CD34 and CD38. (B) KG-1a and MOLM13 cells were enriched in LSCs (9.6% and 4.3%, respectively). (C) Flow cytometry analysis for P-gp expression of CD34+CD38-, CD34+CD38+, CD34-CD38+, CD34-CD38- in KG-1a cells (D) Serum-free culture of LSCs from KG-1a cell. (E) Stem-cell-related genes and drug resistance gene (ABCB1) were detected by qPCR (^*^ *P*<0.05). (F) The self-renewal ability of LSCs was detected within soft agar assay, and re-stained for CD34-PE/CD38-FITC in 4 subpopulations after 14 days by flow cytometry. Additional file 2: Figure S2. The LSCs property of drug-resistance. (A) LSC displayed more chemo-resistance ability than Bulk cells with the three first-line AML chemotherapeutics Ara-C, Dexamethasone (Dex) and L-Asparaginase (L-Asp). (B) KG-1a-LSCs and Non-LSC were treated with Arc-C and Dex in the presence of Verapamil (^*^ *P*<0.05, ^**^ *P*<0.01). (C) The columnar statistical chart were analyzed for the apoptosis rates in the cytotoxicity tests. Additional file 3: Figure S3. The protein micro-assay slides and siRNA silencing efficiency. (A) The Human Apoptosis Antibody Array G Series. (B) The siRNA silencing efficiency of Hsp70, survivin, XIAP and IGF-II were verified by Western-blottig, and highest silence sequences were listed below the band. (C) Ectopic expression of pcDNA-Sp1/c-Myc vector was detected by western blot, and GAPDH was used as internal control. Additional file 4: Table S1.
**Primers for amplification of survivin promoter region.** **Table S2.** Primers for mutation in survivin promoter. **Table S3.** Chip Primers and qPCR Primers. Additional file 5: Figure S4. Inhibiting of Sp1 and C-myc caused the chemo-resistance of LSCs. (A-B) MITA could significantly increase the apoptosis rate induced by Ara-C in both cell lines. (C) MITA could obviously reduce the colony numbers of LSC in tumor-sphere formation assay (^**^ *P*<0.01). (D) MITA treatment repressed the core promoter activity of V5 plasmid of the survivin promoter (^**^ *P*<0.01). KG-1a and MOLM13-LSCs were co-transfected with both pGL4-V5(-218/+170) and *Renilla* luciferase plasmid for 12 h, exposed to 200 nM MITA for 48 h, and then subjected to luciferase assays.

## Abbreviations

FAB: French-American-Britain subtype
WBC: White blood cell
LDH: Lactate dehydrogenase
BM: Bone marrow
PB: Peripheral blood. *Fisher’s exact test was used
