# Supplementary figures and images for: Sp1 and c-Myc modulate drug resistance of leukemia stem cells by regulating survivin expression through the ERK-MSK MAPK signaling pathway
Source: Mol Cancer. 2015 Mar 7;14:56. doi: 10.1186/s12943-015-0326-0 (PMC4357193; doi:10.1186/s12943-015-0326-0)

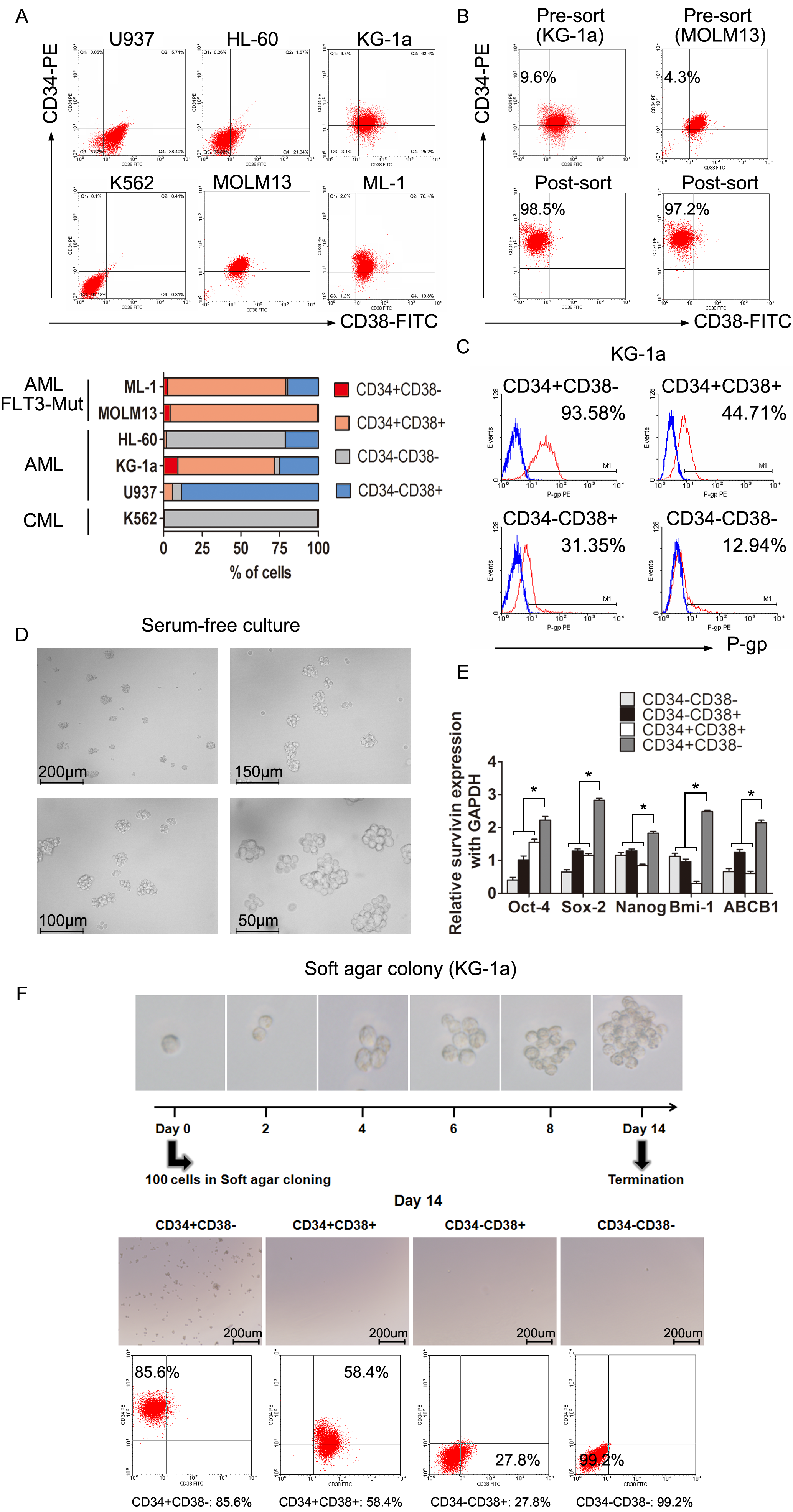

Supplement: Additional file 1: Figure S1. — Isolation and Identification of LSCs. (A) Six leukemia cell lines were selected and identified with the surface marker of CD34 and CD38. (B) KG-1a and MOLM13 cells were enriched in LSCs (9.6% and 4.3%, respectively). (C) Flow cytometry analysis for P-gp expression of CD34+CD38-, CD34+CD38+, CD34-CD38+, CD34-CD38- in KG-1a cells (D) Serum-free culture of LSCs from KG-1a cell. (E) Stem-cell-related genes and drug resistance gene (ABCB1) were detected by qPCR (* P<0.05). (F) The self-renewal ability of LSCs was detected within soft agar assay, and re-stained for CD34-PE/CD38-FITC in 4 subpopulations after 14 days by flow cytometry. [file 12943_2015_326_MOESM1_ESM.tiff]

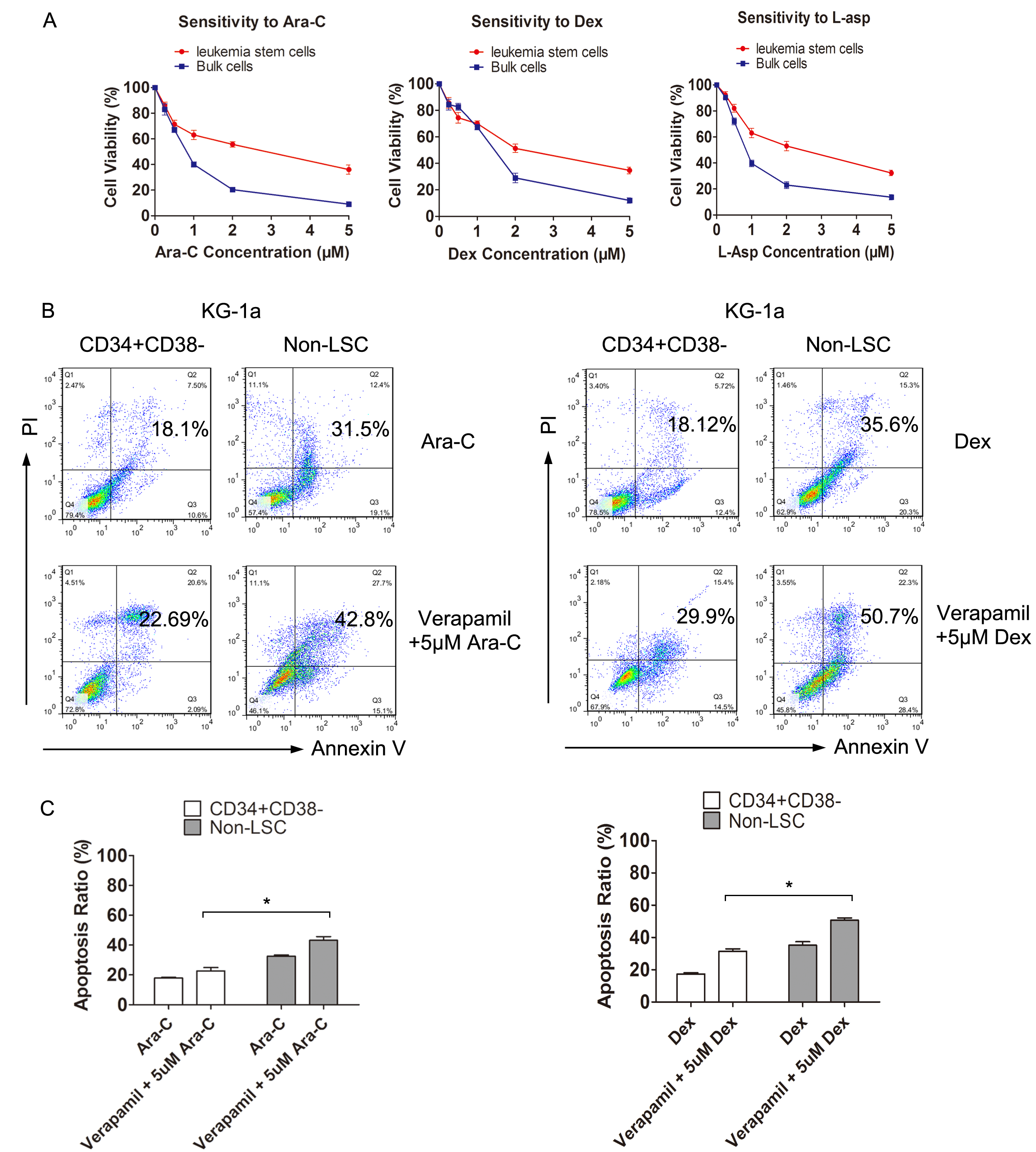

Supplement: Additional file 2: Figure S2. — The LSCs property of drug-resistance. (A) LSC displayed more chemo-resistance ability than Bulk cells with the three first-line AML chemotherapeutics Ara-C, Dexamethasone (Dex) and L-Asparaginase (L-Asp). (B) KG-1a-LSCs and Non-LSC were treated with Arc-C and Dex in the presence of Verapamil (* P<0.05, ** P<0.01). (C) The columnar statistical chart were analyzed for the apoptosis rates in the cytotoxicity tests. [file 12943_2015_326_MOESM2_ESM.tiff]

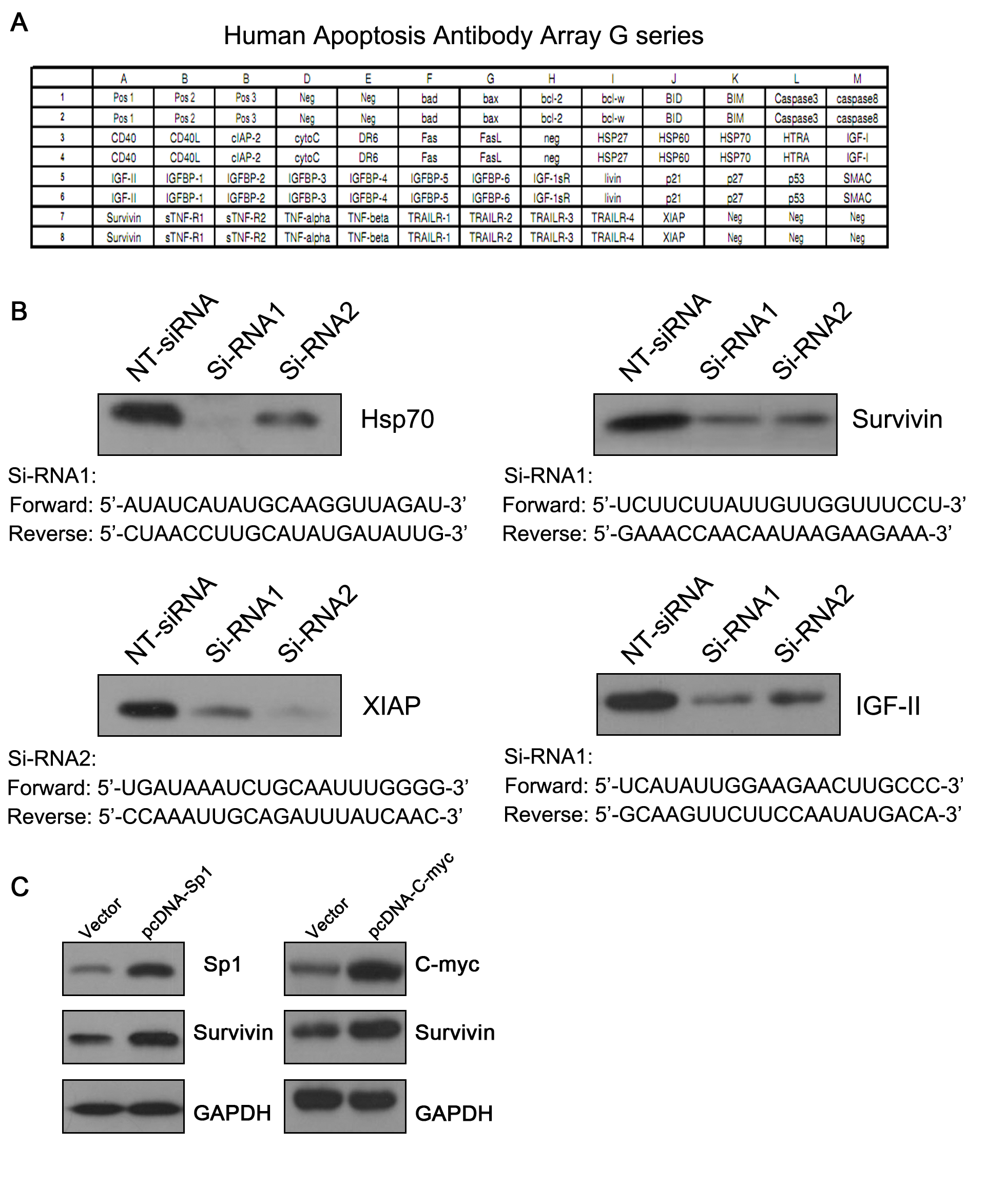

Supplement: Additional file 3: Figure S3. — The protein micro-assay slides and siRNA silencing efficiency. (A) The Human Apoptosis Antibody Array G Series. (B) The siRNA silencing efficiency of Hsp70, survivin, XIAP and IGF-II were verified by Western-blottig, and highest silence sequences were listed below the band. (C) Ectopic expression of pcDNA-Sp1/c-Myc vector was detected by western blot, and GAPDH was used as internal control. [file 12943_2015_326_MOESM3_ESM.tiff]

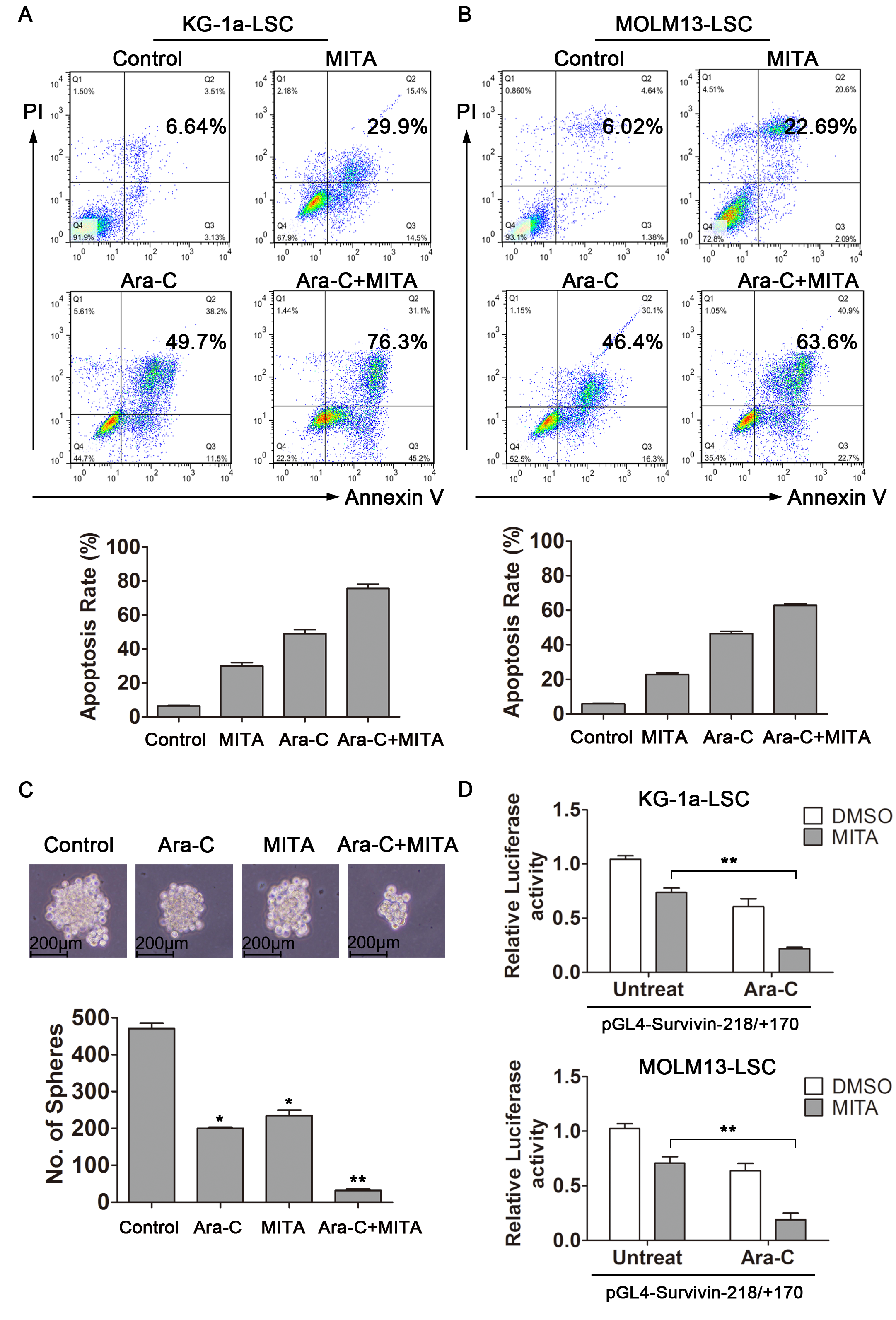

Supplement: Additional file 5: Figure S4. — Inhibiting of Sp1 and C-myc caused the chemo-resistance of LSCs. (A-B) MITA could significantly increase the apoptosis rate induced by Ara-C in both cell lines. (C) MITA could obviously reduce the colony numbers of LSC in tumor-sphere formation assay (** P<0.01). (D) MITA treatment repressed the core promoter activity of V5 plasmid of the survivin promoter (** P<0.01). KG-1a and MOLM13-LSCs were co-transfected with both pGL4-V5(-218/+170) and Renilla luciferase plasmid for 12 h, exposed to 200 nM MITA for 48 h, and then subjected to luciferase assays. [file 12943_2015_326_MOESM5_ESM.tiff]
