# Supplementary material for: Sp1 and c-Myc modulate drug resistance of leukemia stem cells by regulating survivin expression through the ERK-MSK MAPK signaling pathway
Source: Mol Cancer. 2015 Mar 7;14:56. doi: 10.1186/s12943-015-0326-0 (PMC4357193; doi:10.1186/s12943-015-0326-0)
Supplement: Additional file 4: Table S1. — Primers for amplification of survivin promoter region. Table S2. Primers for mutation in survivin promoter. Table S3. Chip Primers and qPCR Primers. [file 12943_2015_326_MOESM4_ESM.docx]

**Table S1. Primers for amplification of survivin promoter region**

| **Construct** **Forward Prime** (5’- to 3’) **Reverse Prime** (5’- to 3’) |
| --- |
| V1(-1935/+170) CCCAAGCTTTTCCTTTCCTCCCTCCTGA CCGCTCGAGTTCAAATCTGGCGGTTAATG  V2(-1589/+170) CCCAAGCTTTAGGGGAGAGAAAGTAGGTT CCGCTCGAGTTCAAATCTGGCGGTTAATG  V3(-1045/+170) CCCAAGCTTAAGAATGGGGGCGGGGTGG CCGCTCGAGTTCAAATCTGGCGGTTAATG  V4(-516/+170) CCCAAGCTTTTTTAGACTGAGTTTTGCTCTT CCGCTCGAGTTCAAATCTGGCGGTTAATG  V5(-218/+170) CCCAAGCTTTGTATTTTTAGTAGAGACAAGG CCGCTCGAGTTCAAATCTGGCGGTTAATG  V6(-1935/-359) CCCAAGCTTTTCCTTTCCTCCCTCCTGA CCGCTCGAGGGCTGAGGCAGGAGAATCG  V7(-1935/-1045) CCCAAGCTTTTCCTTTCCTCCCTCCTGA CCGCTCGAGATTTGAAACTGAGTCTCGC |
| **Table S2. Primers for mutation in survivin promoter**   \| **Construct Primer sequences (5’-3’)** \| \| \| --- \| --- \| \| TCF4 (A) MT  Sp1 (B) MT  KLF5 (C) MT  Sp1 (D) MT  C-myc (E) MT  Sp1 (F) MT  Sp1 (G) MT  Sp1 (H) MT  Sp1 (I) MT  Sp1 (J) MT \| F: CGGCCTGCACGCGTTC***GAC***GAAAGCAGTCGAG  R: CCCCCTCGACTGCTTTCGTCGAACGCGTGCAG  F: AGGGGGCGCTAGGTGT***TAT***CAGGGACGAGCT  R: GCGCCAGCTCGTCCCTGATAACACCTAGCGC  F: TGGCGCGGCGTCGCT***AATA***GCACCGCGACCA  R: GCCCGTGGTCGCGGTGCTATTAGCGACGCCGC  F: CACCGCGACCACGG***TAC***GAGCCACGCGGCGG  R: CCTCCCGCCGCGTGGCTCGTACCGTGGTCGC  F: ACGGGCAGAGCC***GGA***CGGCGGGAGGACTAC  R: GTAGTCCTCCCGCCGTCCGGCTCTGCCCGT  F: ACGGGCAGAGCCACGC***ATT***GGGAGGACTAC  R: GTAGTCCTCCCAATGCGTGGCTCTGCCCGT  F: GGAGGACTACAACTCCC***TAG***ACACCCCGCG  R: CGCGGGGTGTCTAGGGAGTTGTAGTCCTCC  F: ACACCCCGCGCCG***TTAA***GCCTCTACTCCCA  R: TGGGAGTAGAGGCTTAACGGCGCGGGGTGT  F: GGGGGTGGACC***ATA***TAAGAGGGCGTGCGCT  R: AGCGCACGCCCTCTTATATGGTCCACCCCC  F: CGCCTAAGAGGG***ACA***GCGCTCCCGACATGC  R: GCATGTCGGGAGCGCTGTCCCTCTTAGGCG \| |

**Table S3. Chip Primers and qPCR Primers**

| **Name Forward Prime** (5’- to 3’) **Reverse Prime** (5’- to 3’) | | |
| --- | --- | --- |
| **Primers for ChIP**  **Binding region** | | AGCGCACGCCCTCTTAGGCG  GGGGGCAACGTCGGGGCACC  ACCGGATCGCATTCGGATTATC  CCGGCTACGATCGCAGTCCAG  AAGTCTGGCTCGTTCTCAGTG  CCGTATATGTCTACACACAGATG  TGTTCGCCTCTTGACATTCTC  TCGCTCCTGGAAGATGGTGAT |
| -218/-15 C-myc  -117/+89 Sp1  **Distal region**  -2783/-2634 C-myc  -2783/-2634 Sp1  **Primers for qPCR**  Survivin  Sp1  C-myc  GAPDH | GCCGGACGTGCGCAAGAAAC  GGTGCGCCGCCCTCCTGATG  CGTACCGCTACAGGCCTCCA  CCGCATGCGGATACCATTCCG  CACCGCATCTCTACATTCAAGA  AAGAAATGACCTTAGGAACATAC  CCTCCACTCGGAAGGACTATC  AACGGATTTGGTCGTATTGGG |  |
